# Absence of *BRCA*/*FMR1* Correlations in Women with Ovarian Cancers

**DOI:** 10.1371/journal.pone.0102370

**Published:** 2014-07-18

**Authors:** Norbert Gleicher, Jessica N. McAlpine, C. Blake Gilks, Vitaly A. Kushnir, Ho-Joon Lee, Yan-Guang Wu, Emanuela Lazzaroni-Tealdi, David H. Barad

**Affiliations:** 1 Center for Human Reproduction, New York, New York, United States of America; 2 Foundation for Reproductive Medicine, New York, New York, United States of America; 3 Division of Gynecologic Oncology, Department of Gynecology and Obstetrics, University of British Columbia, Vancouver, British Columbia, Canada; 4 OvCaRe Gynecologic Tissue Bank, BC Cancer Agency, Vancouver, British Columbia, Canada; 5 Department of Pathology and Laboratory Sciences, University of British Columbia, Vancouver, British Columbia, Canada; University of South Alabama Mitchell Cancer Institute, United States of America

## Abstract

Previously reported findings in Austrian *BRCA1*/2 mutation carriers suggested a possible dependency of embryos with *BRCA1*/2 mutations on so-called *low* alleles of the fragile X mental retardation 1 (*FMR1*) gene, characterized by less than 26 CGG repeats (CGG_n<26_). The hypothesis arose from a study reporting highly statistically significant enrichment of *low FMR1* alleles, significantly exceeding *low* allele prevalence in a general population, suggesting embryo lethality of *BRCA1*/2 mutations, “rescued” by presence of *low FMR1* alleles. Such a dependency would also offer an explanation for the so-called “*BRCA*-paradox,” characterized by *BRCA1*/2 deficient embryonic tissues being anti-proliferative (thereby potentially causing embryo-lethality) but proliferative in malignant tumors, including breast and ovarian cancers. Follow up investigations by other investigators, however, at most demonstrated trends towards enrichment but, mostly, no enrichment at all, raising questions about the original observation and hypothesis. We in this study, therefore, investigated CGG_n_ of the *FMR1* gene of 86 anonymized DNA samples from women with various forms of ovarian cancer, and were unable to demonstrate differences in prevalence of *low FMR1* alleles either between positive and negative ovarian cancer patients for *BRCA1*/2 or between ovarian cancer patients and reported rates in non-cancer populations. This raises further questions about a suggested dependency between *BRCA1/2* and *FMR1*, but also raises the possibility that investigated Austrian *BRCA1*/2 carrier populations differ from those in other countries. Either only selected *BRCA1*/2 mutations, therefore, interact with *low FMR1* alleles or the Austrian data reflect only coincidental observations.

## Introduction

Austrian colleagues and we previously reported in an Austrian population of women with functional *BRCA1* and *BRCA2* mutations statistically highly significant enrichment with so-called *low* fragile X mental retardation1 (*FMR1*) gene alleles [1], [2]. Such *low* alleles are defined by less than 26 CGG repeats (CGG_n<26_), and have been associated with premature declines in functional ovarian reserve, also called premature ovarian aging (POA) or occult primary ovarian insufficiency (OPOI) [3].

Since *BRCA1* mutations have also been associated with POA/OPOI [4], above described findings in Austrian *BRCA1/2* mutation carriers led to the hypothesis that *BRCA1* effects on ovarian function may actually reflect *FMR1* effects. Under this hypothesis, *BRCA1/2* mutations are, in principle, embryo-lethal [1], a suggestion supported by some homozygous *BRCA1*/2 mouse homologs, indeed, being embryo-lethal, though with considerable variability in phenotype and in rescue from lethality on a p53-null background [5]. Embryos so, potentially, destined for mortality, if also carrying *low FMR1* alleles, would, however, be rescued, leading to the enrichment of *low FMR1* alleles in now rescued carriers of *BRCA1*/2 mutations, as observed in Austrian women [1], [2].

This hypothesis also, for the first time, offered an explanation for the so-called “*BRCA* paradox,” which received its name from the contradictory observations that *BRCA1/2* deficient tumor cells very rapidly proliferate, while *BRCA1/2*-deficient embryos suffer from proliferation defects (and, possibly, therefore succumb to embryo lethality) [5]. In animal models, p-53-nullizygosity can rescue *BRCA1* mouse mutant but, often, only delays lethality [6]–[10].

In humans, *BRCA1*/2 mutations are strongly associated with increased risk for malignancies, including breast and ovarian cancers [11]. If *low FMR1* alleles were to be able to suppress anti-proliferative (and, therefore, embryo-lethal) effects of *BRCA1/2* mutations, allowing carriers of *low FMR1* mutations to escape embryo-lethality, only *BRCA1*/2 carrying embryos would be born. They also would carry a *low FMR1* allele, and grow up with suppressed anti-proliferative effects (i.e., would express a proliferative phenotype) and, therefore, be at risk for *BRCA1*/2-associated cancers. The actual culprit for cancer risk under such a scenario would, therefore, actually be the suppressive effect of *low FMR1* alleles on *BRCA1*/2, converting anti-proliferative into a proliferative phenotypes [1].

The potential importance of this hypothesis for oncology attracted follow up by investigators in The Netherlands [12], Israel [13] and Italy [14]. All three studies, however, failed to confirm the Austrian observation of *low FMR1* allele enrichment amongst carriers of *BRCA1*/2 mutations. As a possible explanation, we noted in an accompanying editorial to the Italian study that investigated *BRCA1/2* mutations in Austrian and Italian study patients were completely different [15].

Divergent results between Austrian and Italian studies, therefore, could reflect different *BRCA1*/2 mutations with different degrees of embryo lethality. *BRCA1*/2 mutations in these two countries are, indeed, known to diverge [16]. Especially relevant to the Dutch study [12], Verhoog et al reported that even within The Netherlands, significant divergence in *BRCA1*/2 mutations is observed even within very small geographic areas [17]. Finally, the Israeli study involved practically exclusively *BRCA1*/2 founder mutations associated with cancer risk in Ashkenazi Jewish populations [13] and, therefore, was by definition different from *BRCA1*/2 mutations in Austrian populations.

The possibility that different *BRCA1*/2 mutations may exhibit different degrees of dependency with the *FMR1* gene was potentially also supported by the trend towards enrichment with *low FMR1* alleles among *BRCA1*/2 mutation carriers observed in the Italian study (32.6% vs. 23.1%) [14]. Speaking against such an explanation, a recent study, however, suggested other, non-*FMR1*-associated molecular mechanisms as causes for *BRCA1*-associated POA/OPOI [18].

With the issue still unresolved, we, therefore, decided to further explore it in women with ovarian cancer. The hypothesis of here presented study is that, since ovarian cancer risk is associated with *BRCA1*/2 mutations [11], if *low FMR1* alleles, indeed, are causally related to proliferative *BRCA1/2* cancer risks, (i) women with ovarian cancers, overall, should demonstrate a higher prevalence of *low FMR1* alleles than has been reported in cancer-free populations; and (ii) *BRCA1/2*-positive ovarian cancer patients should demonstrate more *low FMR1* alleles than *BRCA1*/2-negative patients.

## Materials and Methods

The study population involved genetic materials from 86 ovarian cancer patients, for who cryopreserved DNA samples were stored at -80°C at the University of British Columbia, Vancouver, Canada.

### IRB approvals and specimens' origin

Material transfer agreements were executed between the University of British Columbia and the Center for Human Reproduction (CHR) in New York City, and approvals for the studies from both Institutional Review Boards (University of British Columbia, Vancouver, Canada, and The Center for Human Reproduction, New York, N.Y.) were separately obtained, including waivers from both IRBs to get individual informed consents from patients who were the source of the genetic materials investigated because samples were coded, before the specimens were shipped overnight on dry ice from Vancouver to New York City. Each specimen contained at least 100 ng of DNA in 5 to 10 uL volume.

### Illumina sequencing of CCGn

The exonic and limited flanking intronic sequence of *BRCA1*/2 was determined from peripheral blood derived gDNA following amplification using RainDance technology and Illumina sequencing. The resulting sequences were aligned to the hg19 human genome reference using BWA (both aln and bwasw algorithms), and assembled with ABySS. Variant calling was performed using the samtools mpileup (ABySS, bwasw, and aln) and pindel (aln only) packages. Identified variants were submitted by report to CGL. CGL: Submitted variants were interpreted and annotated using HGVS nomenclature, using reference sequences NM_007294 for *BRCA1*, and NM_000059 for *BRCA2*. Pursuant to HGVS convention, cDNA numbering begins at the A of the initiating codon (ATG). Sequences of low coverage regions and ACMG category 1 and 2 mutation variants were confirmed by Sanger sequencing. This test was developed and its performance characteristics determined by the Centre for Clinical Diagnostic Genomics and further validated at the Cancer Genetics Laboratory (BCCA).

### MLPA

The presence or absence of copy number differences in *BRCA1*/2 genes or portions thereof, were determined via Multiplex Ligation-dependant Probe Amplification (MLPA) according to the manufacturer's protocol (P002-C1, P090-A3, MRC-Holland, Amsterdam). Analysis of the resulting amplification products was performed using an ABI 3730 DNA Analyzer and associated analysis software. Large scale insertions and deletions which lie outside the regions assessed by the individual MLPA probes are not detectable by this method. Genetic variants lying within individual probe binding sites may lead to false positive MLPA results. Single exon deletions are independently confirmed. *BRCA1* reference sequence: NM_007294. *BRCA2* reference sequence NM_000059.

This test was developed and its performance characteristics determined by MRC-Holland (Amsterdam). Furthermore, this test kit is labeled “For Research Purposes Only.”

Specimens were initially shipped anonymized with identifier codes. Once *FMR1* testing results had been obtained, clinical information in regards to each sample was forwarded from Vancouver to New York, which included *BRCA1* and *BRCA2* status, type of ovarian malignancy and stage of disease.

Once specimens were received in New York, they were immediately stored at −80°C until assayed by commercial assay for CGG_n_ of the *FMR1* gene (LabCorp, Burlington, North Carolina), as previously reported [3]. In short, no interpretable results were obtained in 6/86 submitted samples, leaving 80 ovarian cancer patients in the study for analysis. CGG_n_ was reported for both alleles. Individual mutations were described as previously reported based on a normal CGG_n_ range of 26–34. Alleles below CGG_n = 26_ were, therefore, considered *low* [3]. Women with both alleles in normal range are considered normal (*norm*); those with one allele in normal and one outside normal range are heterozygous (*het*) and those with both alleles outside normal range are homozygous (*hom*). Genotypes are then further sub-divided into sub-genotypes based on *low* or *high* (CGG_n>34_) alleles.

We then established the prevalence of *low FMR1* alleles for the whole ovarian cancer group and compared it to control populations without known malignancies, previously reported in the literature. In a second analysis we then compared the prevalence of *low FMR1* alleles in ovarian cancer patients, either with or without *BRCA1*/2 mutations. And, in addition, repeated the analysis only for functionally oncogenic *BRCA1*/2 mutations.

Statistical analyses were performed using IBM SPSS statistics version 21. Continuous variables were expressed a means ± standard deviation. Categorical variables were expressed as counts (percentage). Results were cross-tabulated and Chi Square test was used to compare different distributions.

## Results

Satisfactory *FMR1* results were obtained from 80/86 samples. Table 1 summarizes patient characteristics for these 80 patients.

**Table 1:** Ovarian cancer patient characteristics.

Characteristic	Detail	n = 80^1^	Percent
*FMR1*			
	*norm*	48	60.0%
	*het-norm/high*	8	10.0%
	*het-norm/low*	19	23.8%
	*hom* *	5	6.3%
			
Ovarian cancer diagnosis			
	High-grade serous	60	75.0%
	Clear cell	9	11.3%
	Endometroid	6	7.5%
	Low-grade serous	5	6.3%
			
Functional oncogenic *BRCA*			
	*BRCA1*	11	13.8%
	*BRCA2*	4	5.0%
	Negative	65	81.3%
*All BRCA* mutations			
	*BRCA1*	21	26.3%
	*BRCA2*	6	7.5%
	Negative	53	66.3%

1 For 6 cancer patients no FMR1 data were obtainable from submitted samples;

*hom sub-genotypes are not broken out; 4/5 contained *low* alleles.

**Figure 1:**
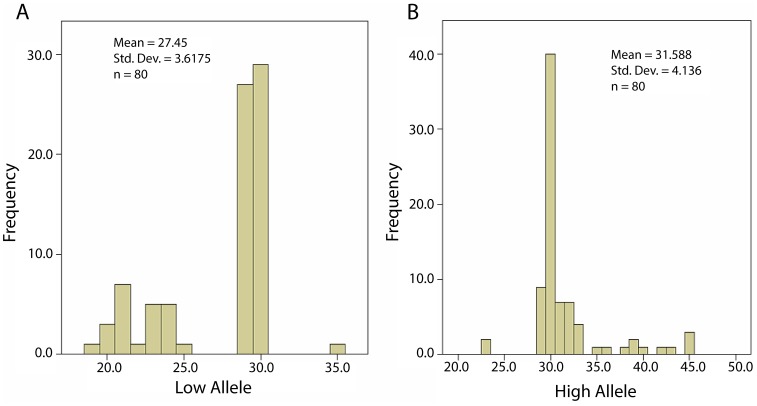
Distribution of CGG_n_ for each ovarian cancer patient's lower and higher *FMR1* Allele (A & B).

Figures 1a (lower *FMR1* allele) and 1b (higher allele) demonstrate the CGG_n_ distribution for the whole patient cohort (means 27.45±3.62, and 31.59±4.14 CGGs, respectively).

Using previously noted abbreviations for normal (*norm*), heterozygous (*het*) and homozygous (*hom*) alleles, Figures 1a and b, thus, primarily demonstrate *norm* genotypes, and more *het-norm/low* than *het-norm/high* sub-genotypes.

**Figure 2:**
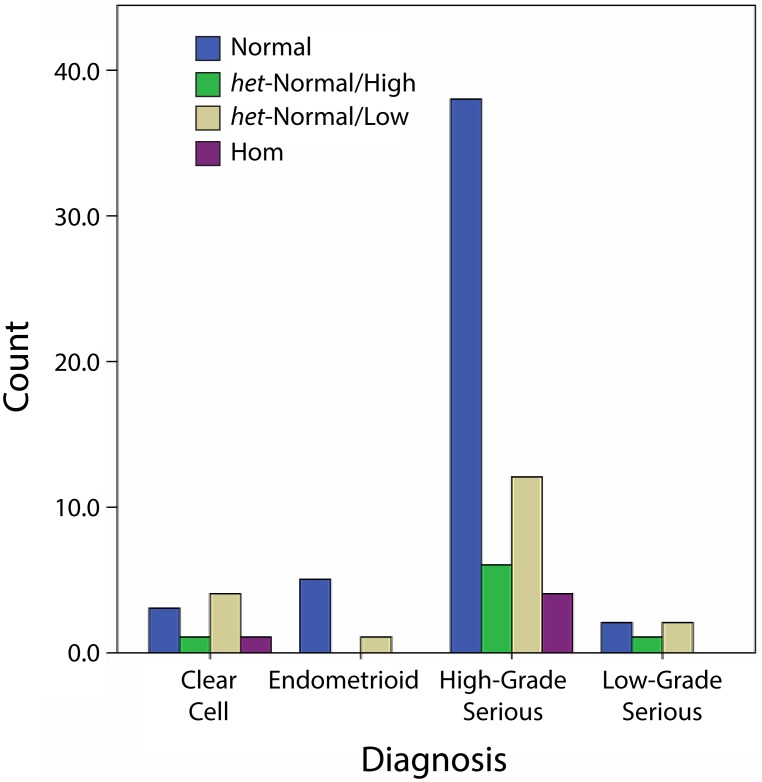
Distribution of *FMR1* genotypes and sub-genotypes in various ovarian cancer types.

A majority of ovarian tumors (60/80, 75.0%) were high-grade serous tumors (Table 1). The remaining in order were clear cell (9/80, 11.3%), endometroid (6/80, 7.5%) and low- grade serous tumors (5/80, 6.3%). These tumor types appeared nominally different in distribution of *FMR1* genotypes/sub-genotypes (Figures 2), but observed differences did not reach statistical significance (Pearson Chi-Square 6.872; df 9, P = 0.65, NS).

Amongst 80 cancer patients for whom *FMR1* data were available, only 27 (33.8%) were *BRCA*-positive, 21 carriers of *BRCA1* and 6 of *BRCA2* mutations. However, amongst *BRCA1*/2 mutations recorded among study group patients, only 15/80 (18.8%) were considered functionally oncogenic. *FMR1* data will, therefore, be presented separately for the whole *BRCA1*/2 population and only functionally oncogenic *BRCA1*/2 mutations.

Table 2 presents the prevalence of *low FMR1* alleles in the ovarian cancer population of this study in comparison to the prevalence reported in the literature for other populations.

**Table 2:** Percent *low FMR1* alleles in ovarian cancer patients and no-cancer cohorts (%).

		Prevalence of *low FMR1* alleles (%)	
**Ovarian cancer patients**	***BRCA1*** **/2-negative**	**18/57**	**31.6**
	*BRCA1*/2-positive	5/23	21.7
**Austrian study ** [1] *	**Infertile female controls**		**20.5**
	*BRCA1*/2-positive		78.8
**Dutch study ** [12] **	*BRCA1*/2-positive		35.0
**Israeli study ** [13]	**Healthy controls**		**31.5**
	*BRCA1*/2-positive		24.8
**Italian study ** [14]	**Healthy controls**		**23.1**
	*BRCA1*/2-positive		32.6

*Reports only *het-norm/low* sub-genotype since did not separately evaluate *low hom* sub-genotypes. True prevalence of *low FMR1* alleles was, therefore even a few percentage points higher.

** Percentage of control population only graphically reported.

The table demonstrates data for all *BRCA1*/2 mutation carriers, whether functionally oncogenic or not. In this group of ovarian cancer patients the prevalence of *low FMR1* alleles was actually nominally higher in *BRCA1*/2-*negative* (18/57, 31.6%) than *BRCA1*/2-positive ovarian cancer patients (5/23, 21.7%; P = 0.43), though the difference did not reach statistical significance.

When the same analysis was repeated for only 15 functionally oncogenic *BRCA1*/2 mutations, outcomes were very similar, 2/15 (13.3%) *low FMR1* alleles in *BRCA1*/2 mutation carriers and 21/65 (32.3%) in ovarian cancer patients without *BRCA1*/2 mutations (P = 0.21).

Both analyses, thus, demonstrate that the combined presence of *BRCA1*/2 mutations and *low FMR1* alleles actually appears to be less commonly associated with ovarian cancer than absence of both of these mutations in the same patient.

As the table further demonstrates, the non-cancer patient populations in the U.S. (controls for the Austrian study) [1] and Italy [14] demonstrated a relative low prevalence of *low FMR1* alleles in the 20.5–23.1% range, while Israeli controls were reported to demonstrate as much a 31.5% *low FMR1* alleles. In contrast, even excluding the 78.8% prevalence of *low FMR1* alleles in the Austrian study (likely even underreported since rare *hom* patients were not sub-divided in that study, thus not including *hom-high/low* and *hom-low/low* patients), the Dutch [12] reported 35.0% prevalence and the Italians [14] a 32.6% prevalence of *low FMR1* alleles. Only the Israeli study [13], therefore, like here reported ovarian cancer data, reported an actually inverted picture of more *low FMR1* alleles in *BRCA1*/2-negative than *BRCA1*/2-positive women. This study, however, was restricted to only 3 predominant founder Ashkenazi mutations for Ashkenazi Jewish populations, in *BRCA1* 185delAG, 5382insC, and 617delT in *BRCA2*.

We previously reported that Austrian [1] and Italian [14] studies did not overlap in any *BRCA1*/2 mutations [15]. As noted above, the Israeli study was restricted to three *BRCA1*/2 founder mutations, predominantly only found in Ashkenazi Jewish populations. [13], and the Dutch study did not report *BRCA1*/2 mutations in their study population [12], though others reported very significant regional differences in *BRCA1*/2 mutations even within this relatively small country [17]. The individual *BRCA1*/2 mutations in here reported ovarian cancer patients are reported in the Table 3, and also demonstrated no significant overlap with either Austrian or Italian studies.

**Table 3:** *BRCA1*/2 mutations in here presented ovarian cancer patients.

	*BRCA1/2* mutations		
	*HGVS*		*BIC*
***BRCA1***			
		**Undefined 3**	
			**2250A>T**
	**c.3302G>a**		**1048delA**
	**c.422-?**		**547+?del**
	**c.4186-?**		**4357+?dup**
	**c.6406>T**		**-**
	**-**		**1048delA**
	**-**		**3726C>T**
	**-**		**4184delTCAA**
	**-**		**185delAG**
	**-**		**4797G>T**
	**-**		**5370C>T**
	**-**		**546G>T**
	**c.3758C>G** 1		**?**
	**c.1530A>C**		**pending**
	**c.5236C>G**		**-**
	**c.4812A>G**		**-**
	**c.4039A>G**		**-**
	**c.4883T>C**		**-**
	**c.548-17T>G**		**28146T>G**
	**c.3328_3330delAAG**		**-**
			
***BRCA2***			
	**c.2883G>A** 1		**?**
	**c.2808_2811delACAA**		**-**
	**c.4848-4849delAA**		**-**
	**-**		**5445delTTTAAGT**
	**c.4715C>G**		**-**
	**c.7301A>C**		**-**
	**c.4314C>T** 1		**-**

1 Two patients were carriers of *BRCA1* and *BRCA2* mutations;

## Discussion

We in this study investigated in women with various forms of ovarian cancer whether the presence of *BRCA1*/2 mutations resulted in enrichment of *low FMR1* mutations, which would suggest interplay between these two genes, in establishing oncogenic risk. We, however, were unable to detect any difference in distribution of *low FMR1* alleles in comparison to reported distributions in normal infertile populations without known malignancies [1], [12]–[14], nor were we able to demonstrate a relative increase in *low FMR1* alleles in *BRCA1*/2 carriers with ovarian cancers in comparison to ovarian cancer patients who were not *BRCA1*/2 mutation carriers.

Indeed, this study actually demonstrated the opposite, a normal-range prevalence of *low FMR1* alleles in *BRCA1*/2 mutation-carrying ovarian cancer patients but a trend towards higher prevalence in ovarian cancer patients who were not *BRCA1*/2 carriers. Interestingly, a similar result was reported in the Israeli study [13], where *BRCA1*/2 mutation carriers, a large majority of them already diagnosed with breast cancer, demonstrated only in 24.8% *low FMR1* alleles, while random controls demonstrated *low FMR1* alleles in 31.5% of women.

Why here reported ovarian cancer patients without *BRCA1*/2 mutations and Israeli controls present with such an unusually high, and apparently elevated prevalence over average populations, of *low FMR1* alleles is unclear. In a large majority, *low FMR1* alleles represent *het-norm/low FMR1* sub-genotypes. In a small minority they also can represent either *hom-high/low* or *hom-low/low* sub-genotypes. Combined, low alleles rarely represent more than approximately 25% of an infertile female population [3].

Here reported findings, however, do offer some potentially important answers: They make the hypotheses increasingly unlikely that (i) all *BRCA1*/2 mutations in humans are to a significant degrees embryo-lethal; (ii) *low FMR1* alleles rescue embryos from *BRCA*-lethality and (iii) the *FMR1* gene offers a final solution to the “*BRCA* paradox.”

Considering that hundreds of *BRCA1/2* mutations have been reported, amongst which only few are functionally associated with increased cancer risks, even considering here presented study results, one, however, still cannot preclude that the previously suggested hypothetical interplay between *BRCA1*/2 and *FMR1* genes, similarly, may be only restricted to selected *BRCA1*/2 mutations.

Such an explanation would suggest that the Austrian study, which so strongly suggested an embryonic selection process for *low FMR1* alleles, disproportionally reflected a selective embryo-lethal *BRCA1*/2 population, favoring interaction with the *FMR1* gene. Otherwise, this study of Austrian patients would have to be considered a statistical coincidence, though conducted in blinded fashion, with all *BRCA* and *FMR1* assays performed in Austria by well established genetic laboratories in academic centers, while statistical analysis of assay data was, independently, performed in the U.S. [1].

While here reported study, therefore, further diminishes the likelihood that the *BRCA* and *FMR1* genes interact in their effects on embryo survival and oncogenic risk, the study does not preclude the possibility that selected embryo-lethal oncogenic mutations of *BRCA1/2*, indeed, are rescued by *low FMR1* alleles.

In this context, it is interesting to note that a variety of genome-wide association studies of *BRCA1/2* mutation carriers recently identified some genetic loci, which affect *BRCA1*/2-associated cancer risks for breast and ovarian cancers [19]–[21]. The thought that specific mutations of the *FMR1* gene may, selectively, affect *BRCA1*/2, therefore, is conceivable.

*BRCA* is generally considered a genetic repair gene, which, when mutated, amongst other negative effects, can also affect X-chromosome inactivation [22]. Skewed activation in women with breast and ovarian cancers, at least in part, has been attributed to *BRCA1* and to a lesser extend *BRCA2* mutations [23].

One also can further hypothesize about potential bi-directional effects of these two genes on each other. For example, certain *BRCA1*/2 mutations could affect the *FMR1* gene, located at Xq27.3, via X-chromosome inactivation and methylation of *FMR1*. The *FMR1* gene, in turn, could rescue, as previously hypothesized [1], selected embryo lethal *BRCA1*/2 mutations. Such interactive effects between the two genes would, of course, result in much more complex clinical phenotypes. Studies like this or previously reported studies by others [12]–[14], therefore, likely would not be able to discover such interactions between the two genes.

An *FMR1* interaction as explanation of the “*BRCA* paradox,” therefore, appears increasingly unlikely but still cannot be completely excluded.

This study for the first time investigated the alleged *BRCA1/2* interaction with *low FMR1* mutations in an ovarian cancer model. All prior studies were conducted in breast cancer patients. The use of another *BRCA1/2* associated cancer model, and the quite large number of available patient samples represent the strengths of this study. Somewhat of a weakness lies in the absence of racial data on investigated patients since *FMR1* mutation prevalence to a degree is racially defined [24]. Ontarian law, however, does not allow for maintenance of such data in association with genetic studies.
